# Durability of Spike Immunoglobin G Antibodies to SARS-CoV-2 Among Health Care Workers With Prior Infection

**DOI:** 10.1001/jamanetworkopen.2021.23256

**Published:** 2021-08-30

**Authors:** Emily R. Egbert, Shaoming Xiao, Elizabeth Colantuoni, Patrizio Caturegli, Avinash Gadala, Aaron M. Milstone, Amanda K. Debes

**Affiliations:** 1 Johns Hopkins University School of Medicine, Baltimore, Maryland; 2 Johns Hopkins School of Public Health, Baltimore, Maryland

## Abstract

This cohort study examines the durability of spike antibodies to SARS-CoV-2 among a cohort of US health workers.

## Introduction

Herd immunity is needed to reduce deaths, prevent transmission, and minimize the emergence of SARS-CoV-2 variants. The durability of serum antibodies against the spike protein of this virus provides insights into immunologic memory following natural infection.^1^ Recent literature supports that the levels of spike antibodies induced by natural infection correlate with neutralization and protect against subsequent infection.^2^ We evaluated the durability of naturally acquired spike immunoglobin (Ig) G antibodies to SARS-CoV-2 among a cohort of health care workers.

## Methods

Beginning in June 2020, 3015 hospital workers (HWs) at 5 regional hospitals in the Johns Hopkins Health System consented to participate and were enrolled in a prospective cohort study to determine the seroprevalence of spike antibodies to SARS-CoV-2. This study was approved by the Johns Hopkins University institutional review board. Cohort results followed the Strengthening the Reporting of Observational Studies in Epidemiology (STROBE) reporting guideline. Data were analyzed March 2021.

Participants provided serum samples and completed surveys (including providing demographic data and exposures) every 3 to 4 months after enrollment. SARS-CoV-2 polymerase chain reaction (PCR)–testing and immunization data were collected from electronic health records. A convenience sample of HWs who tested positive for SARS-CoV-2 and then had at least 1 positive anti–SARS-CoV-2 IgG measurement prior to vaccination were included in this analysis. Serum specimens were tested using an enzyme-linked immunosorbent assay (Euroimmun) that targets the S1 subunit of the SARS-CoV-2 spike protein and measures optical density ratios. We applied an internally derived IgG cutoff ratio (>1.23) for greater sensitivity and specificity with an upper threshold of 11 based on assay saturation.^3,4^

Median serum IgG ratios as a function of time (ie, days from positive PCR test) were visualized using a natural cubic spline (with 2 *df*) with 95% bootstrap CIs to account for multiple serum samples within HWs. A linear mixed model with random intercept for each HW quantified the relative change in serum IgG ratio per day from a positive PCR test. A sensitivity analysis, including only HWs with multiple serum samples, estimated the within-participant relative change in IgG by separating the cross-sectional and longitudinal effect of time. Analysis was conducted using R version 4.0.2 (R Project for Statistical Computing). The threshold for statistical significance was α < .05 in 2-sided tests.

## Results

Among the cohort of 3015 HWs (2359 [78.3%] women; median [interquartile range {IQR}] age, 38.4 [31.6-50.0] years), 170 (5.6%) HWs had positive PCR results for SARS-CoV-2, of which only 94 (3.1%) were tested for spike antibodies after infection but before vaccination (57 HWs received 1 antibody test after PCR positive, 36 received 2 tests, and 1 received 3 tests). Of the 94 HWs, 90 (96%) were non-Hispanic/Latino and 70 (74%) were White; the median (IQR) age of HWs tested after PCR-positive results was 37.5 (31.1-46.7) years (Table).

**Table.** Study Cohort Characteristics

Characteristics	HWs, No. (%)		
	All (n = 3015)	PCR-positive (n = 166)	PCR-positive with ≥1 serum sample prior to vaccination (n = 94)
Sex^a^			
Women	2359 (78.3)	137 (82.5)	76 (80.9)
Men	649 (21.5)	29 (17.5)	18 (19.1)
Other	7 (0.2)	0	0
Ethnicity			
Hispanic/Latino	150 (5.0)	8 (4.8)	4 (4.3)
Not Hispanic/Latino	2865 (95.0)	158 (95.2)	90 (95.7)
Race^b^			
American Indian/Alaskan Native	2 (0.1)	0	0
Asian	368 (12.2)	13 (7.8)	9 (9.6)
African American	191 (6.3)	20 (12.0)	13 (13.8)
Hawaiian/Pacific Islander	6 (0.2)	0	0
White	2377 (78.8)	129 (77.7)	70 (74.5)
Other	71 (2.4)	4 (2.4)	2 (2.1)
Age, median (IQR), y	38.4 (31.6-50.0)	37.1 (31.5-47.2)	37.5 (31.1-46.7)
Age groups			
≤29 y	566 (18.8)	35 (21.1)	20 (21.3)
30-39 y	1068 (35.4)	60 (36.1)	32 (34.0)
40-49 y	624 (20.7)	38 (22.9)	23 (24.5)
50-59 y	483 (16.0)	27 (16.3)	14 (14.9)
≥60 y	274 (9.1)	6 (3.6)	5 (5.3)

Abbreviations: HW, health worker; IQR, interquartile range; PCR; polymerase chain reaction.

^a^ Demographic data for sex were collected through multiple choice survey questions. Listed options were male, female, and other.

^b^ Demographic data for race were collected through multiple choice survey questions. Other was a listed option on the survey; multiple answers were allowed.

The median spike IgG antibody ratios as a function of days from positive PCR test are shown in the Figure. Fifty-two of 59 (88%), 30 of 40 (75%), and 25 of 33 (76%) HWs who tested less than 100, 100 to 200, and more than 200 days post-PCR were IgG positive, respectively. IgG antibodies were positive in 72% (8 of 11) of those tested more than 250 days postinfection. The estimated rate of IgG decay was 7% per month (95% CI, 3%-10%). In participants with multiple tests postinfection, the within-participant rate of decay was 7% (95% CI, 3%-11%) per month.

**Figure.**
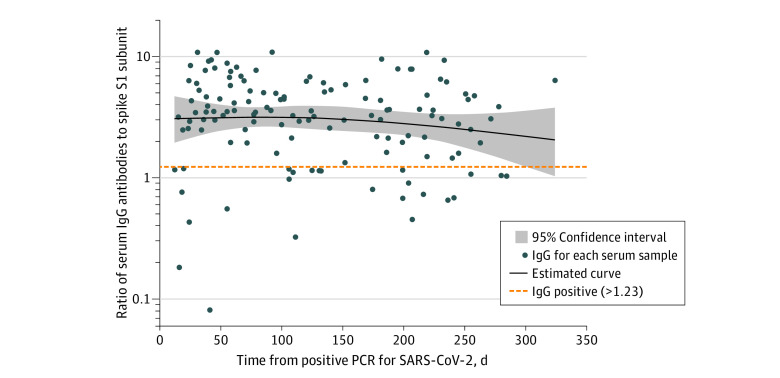
Naturally Acquired Serum IgG Antibodies to SARS-CoV-2 Over Time in Previously PCR Positive HWs Naturally acquired serum immunoglobin (Ig) G antibodies to the S1 domain of the spike protein of SARS-CoV-2 over time in hospital workers (HWs) who previously received positive polymerase chain reaction (PCR) results for SARS-CoV-2. The line represents mean IgG as a function of days from positive PCR test, based on a natural cubic spline (2 *df*). The 95% CI was constructed via 10 000 bootstrap samples of HWs. IgG antibody measurements were determined based on optical density ratios with an upper threshold of 11 based on assay saturation.

## Discussion

Our results demonstrated the durability of spike antibodies to SARS-CoV-2 up to 10 months after natural infection. The Centers for Disease Control and Prevention acknowledges that prior SARS-CoV-2 infection reduces the risk of reinfection for a minimum 90-day period. Our data demonstrate durability of IgG titers well beyond this period and extend recently published intervals of 6 to 8 months.^2,5^

This study was limited by its use of a convenience sample nested within a longitudinal cohort of hospital workers, which means results may not be generalizable. Additional studies are also needed to determine whether antibody levels represent lasting immunity to the evolving SARS-CoV-2.

As vaccine supply remains limited globally, those with naturally derived antibodies may contribute to herd immunity. Therefore, some countries may consider prioritizing vaccination for those without measurable antibodies, a strategy used in prior vaccination campaigns when shortages exist.^6^ However, more research is needed to understand protection against emerging variants based on natural vs vaccine-derived immunity.
